# Asymmetrical habitat coupling of an aquatic predator—The importance of individual specialization

**DOI:** 10.1002/ece3.4973

**Published:** 2019-02-23

**Authors:** Maria H. K. Marklund, Richard Svanbäck, Leanne Faulks, Martin F. Breed, Kristin Scharnweber, Yinghua Zha, Peter Eklöv

**Affiliations:** ^1^ Department of Ecology and Genetics, Limnology, Evolutionary Biology Centre Uppsala University Uppsala Sweden; ^2^ School of Biological Sciences, Department of Ecology and Evolutionary Biology University of Adelaide North Terrace SA Australia; ^3^ Department of Ecology and Genetics; Animal Ecology, Evolutionary Biology Centre Uppsala University Uppsala Sweden; ^4^ Sugadaira Research Station Mountain Science Center University of Tsukuba Ueda Japan; ^5^ Department of Microbiology, Tumor and Cell Biology Karolinska Institutet, NKS BioClinicum Solna Sweden

**Keywords:** diet specialization, food web, landscape genetics, morphological specialization, *Perca fluviatilis*

## Abstract

Predators should stabilize food webs because they can move between spatially separate habitats. However, predators adapted to forage on local resources may have a reduced ability to couple habitats. Here, we show clear asymmetry in the ability to couple habitats by Eurasian perch—a common polymorphic predator in European lakes. We sampled perch from two spatially separate habitats—pelagic and littoral zones—in Lake Erken, Sweden. Littoral perch showed stronger individual specialization, but they also used resources from the pelagic zone, indicating their ability to couple habitats. In contrast, pelagic perch showed weaker individual specialization but near complete reliance on pelagic resources, indicating their preference to one habitat. This asymmetry in the habitat coupling ability of perch challenges the expectation that, in general, predators should stabilize spatially separated food webs. Our results suggest that habitat coupling might be constrained by morphological adaptations, which in this case were not related to genetic differentiation but were more likely related to differences in individual specialization.

## 1 INTRODUCTION

The stability of complex ecosystems depends on the maintenance of the distinct properties of energy channels, such as different levels of productivity and turnover rate (McCann, Rasmussen, & Umbanhowar, 2005; Rooney, McCann, Gellner, & Moore, 2006). Ecological models have shown that mobile predators can help maintain these properties by foraging over a large range of habitats and providing links between the prey items found within them, known as habitat coupling (McCann et al., 2005; Rooney et al., 2006; Rooney, McCann, & Moore, 2008). This might be especially stabilizing in ecosystems with asymmetrical flows of energy, where one energy channel dominates over others in turnover rate and productivity (Rooney et al., 2006). For example, in aquatic systems the productivity of the pelagic food channel is generally higher than the benthic channel, mainly due to the shorter generation time of pelagic compared to benthic primary consumers (i.e., zooplankton vs. benthic invertebrates) (Rooney et al., 2006). In order to stabilize ecosystems with asymmetric flows of energy, mobile predators need to have unequal preferences for prey types, as well as the capacity to rapidly respond to fluctuations in abundance of prey populations, thereby reducing resource variability (McCann et al., 2005). This capacity for a rapid behavioral response to fluctuating resources can depend on intraspecific niche partitioning of the predators (Knudsen, Primicerio, Amundsen, & Klemetsen, 2010; Quevedo, Svanbäck, & Eklöv, 2009). In addition, the ability of predators to couple distinct aquatic habitats can be limited by factors like temperature (Tunney, McCann, Lester, & Shuter, 2014), habitat size and morphometry (Dolson et al., 2009; Eloranta et al., 2015), and interspecific competition (Eloranta, Knudsen, & Amundsen, 2013; Vander Zanden, Casselman, & Rasmussen, 1999). Thus, the dynamics of habitat coupling are complex and may depend on a combination of external as well as population and individual level factors, such as individual specialization.

Individual specialization, where individuals use a small subset of a population's resource base, is common in many animal taxa (Bolnick et al., 2003). The causes of individual specialization can be an individual's preference for resources, food resource use efficiency related to morphology, or their behavioral or physiological ability to handle resources (Smith & Skúlason, 1996; Svanbäck & Eklöv, 2003). In addition, intraspecific competition can affect prey densities and drive individual specialization, leading to variation in net increases in energy, individual fitness, and even fine‐scale genetic population differentiation (Adams et al., 2006; Bolnick et al., 2003; Gerlach, Schardt, Eckmann, & Meyer, 2001; Smith & Skúlason, 1996; Svanbäck & Bolnick, 2005). For example, site fidelity, behavioral traits, spawning time, and kin preference have all been associated with population differentiation over much smaller scales than the dispersal ability of fish (Behrmann‐Godel, Gerlach, & Eckmann, 2006; Gerlach et al., 2001; Smith & Skúlason, 1996). Therefore, the individual specialization of predators has the potential to affect their habitat coupling ability to such an extent that local adaptation and/or genetic differentiation occur.

A predator that is known to exhibit strong dietary and morphological trade‐offs between different habitats is the Eurasian perch (*Perca fluviatilis*; hereafter perch) (Svanbäck & Eklöv, 2003). Perch have a more elongated body in pelagic habitats, where open water favors stronger/faster swimming ability to catch prey, whereas perch in littoral habitats have a deeper body, which is more suited to moving and foraging in complex environments (Eklöv & Svanbäck, 2006; Olsson & Eklöv, 2005; Svanbäck & Eklöv, 2003, 2006). The morphological variation and dietary trade‐offs in perch have been well studied and are largely plastic responses to their environments (Faulks2015a, Svanbäck, Eklöv, & Östman, 2015a; Olsson & Eklöv, 2005; Svanbäck & Eklöv, 2003). If morphological and genetic variations of perch affect their habitat use, then we would also expect these factors to be important for their ability to couple habitats.

Here, we aimed to investigate the associations between individual specialization, morphological adaptations, and/or genetic variation in the predator perch in relation to its ability to couple spatially separate habitats. We define habitat coupling as the ability of perch to feed on more than one spatially separated food web, where the dominance of consumed food resources from only one habitat would indicate low habitat coupling. We consider fluxes among spatially distinct habitats in predator and prey populations, and detritus and nutrients, as habitat linkages (sensu Schindler & Scheuerell, 2002). Due to potential local adaptations of perch, we assume that genetic and morphological adaptations to specific habitats and resources may limit movements between habitats and thereby constrain habitat coupling. Conversely, intraspecific competition can lead to increased individual specialization (IS) on resources that spans across habitats, thereby facilitating habitat coupling. This suggests that habitat coupling is determined by the relative strength of population differentiation constraining habitat coupling and individual specialization promoting habitat coupling. We specifically asked: (a) To what extent do perch couple littoral and pelagic habitats? (b) Are constraints in habitat coupling related to individual specialization, morphological or genetic variation? (c) If habitat coupling is observed, to what degree is it symmetrical? A previous study indicated asymmetric habitat coupling, where littoral perch consumed a substantial amount of pelagic zooplankton whereas the opposite pattern was not found (Scharnweber, Strandberg, Marklund, & Eklöv, 2016). Thus, it is possible that variation in resource preference can lead to asymmetries in habitat coupling.

### 1.1 Methods

### 1.2 Field sampling

**Figure 1:**
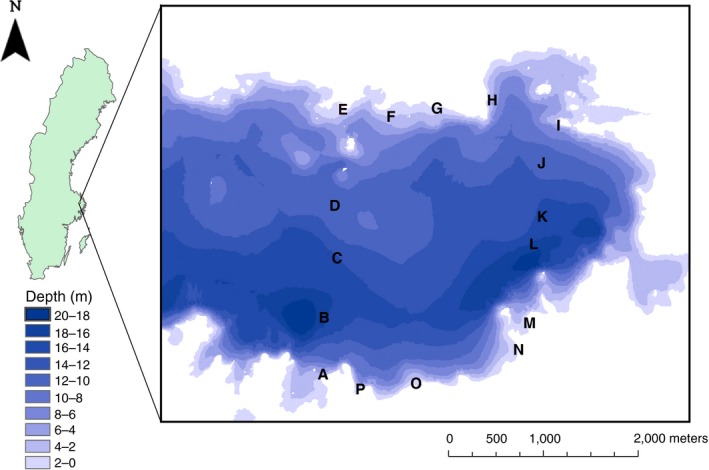
Map of a part of Lake Erken, Sweden, with the locations of sampling sites displayed. Littoral north sites = E, F, G, H, I, littoral south sites = A, M, N, O, P, pelagic west sites = B, C, D, pelagic east sites = J, K, L. © Lantmäteriet Gävle (2012). Permission i2012/921

Perch were sampled from 10 littoral and 6 pelagic sites (Figure 1) in Lake Erken, Sweden (for details of lake characteristics, see: Goedkoop & Pettersson, 2000, Naddafi, Pettersson, & Eklöv, 2010, Pettersson, 1990) in August 2014 using 2 multi‐mesh gill nets of the European standard per site (type Norden; littoral nets 30 × 1.5 m; pelagic nets 27.5 × 6 m). The nets were set in the evening, left for approximately 12 hr, and collected the following morning. To maximize our chances of capturing fish from the littoral zone, nets were set at the lake bottom just outside the reed belt at a water depth of approximately 1.5 m where both the vegetation and the nets extended from the bottom to the water surface. Pelagic nets extended from the water surface and 6 m down in the water column. All fish were frozen immediately after retrieval from the nets and stored frozen until further processing. At each site, 30 perch of 100–140 mm in length were sampled to minimize age cohort variation. This size range of perch is also appropriate to capture niche variation because perch typically undergo two ontogenetic niche shifts and all types of resources used by perch are covered within this size range; zooplankton, macroinvertebrates and fish (Persson, 1988) (see Supporting Information Table S1 for total perch abundance, catch per unit effort, mass and average length measurements for each site). The littoral south region of the lake consisted mostly of soft substrate, whereas the littoral north had a larger proportion of hard rocky substrate, while both pelagic regions consisted of open water. Due to this difference in habitat structure, the 16 lake sites were divided into four regions for statistical analyses: littoral habitat sites were divided into littoral south (five sites) and littoral north (five sites); pelagic habitat sites were divided into pelagic west (three sites) and pelagic east (three sites). In the laboratory, each fish underwent the following procedure: A digital photograph was taken; stomach contents were dissected; muscle tissue was sampled and dried for stable isotope analysis; and a fin clip was taken for genetic analysis (stored in 95% ethanol at −20°C until extraction).

### 1.3 Habitat coupling assessed by habitat‐specific diet and niche use

Stomach contents were analyzed using a dissecting microscope following the approach described in Scharnweber et al. (2016) and Svanbäck and Eklöv (2003). Prey were counted and identified, then classified into nine categories: (a) littoral macroinvertebrates (*Asellus asellus*, *Caenis *spp., Ceratopogonidae, *Cloeon *spp., *Gammarus*, Gastropoda, Hirudinea, *Leptophlebia *spp., Lumbricidae, *Sialis* spp., Trichoptera), (b) benthic Cladocera and Ostracods (*Alona *spp.*, Chydorus *spp.*, Sida *spp.*, *Ostracoda), (c) Chironomidae, (d) pelagic Cladocera (*Bosmina *spp.*, Bytotrephes *spp.*, Daphnia, Polyphemus *spp.), (e) Copepoda (Calanoida, Cyclopoida), (f) pelagic macroinvertebrates (*Chaoborus *spp., Chironomidae pupae, *Leptodora *spp.), (g) fish, (h) crayfish, and (i) terrestrial fauna. The length of the first 10 prey items of each category was measured to the nearest 0.1 mm. The mean length of prey items was converted to biomass (mg dry mass) using our own length–mass regressions, and proportional diet data were calculated for each category and individual. These data were then used to calculate diet indices.

In order to test whether individual variation in niche was related to habitat coupling, we assessed individual specialization (IS), total niche width (TNW), and the between‐individual component (BIC). IS ranges from 1, when all individuals use the full range of the population's niche, to 0, when individuals only consume a single prey type. This means that when the index IS is at 1, individual specialization is at minimum, and when IS is at 0, the specialization is at its maximum and individuals have nonoverlapping diets. TNW is composed of a within and between‐individual component (TNW = WIC + BIC), where the within‐individual component (WIC) is the mean diet variation found within individuals, and the between‐individual component (BIC) is the mean variation in resources used between individuals (Bolnick, Yang, Fordyce, Davis, & Svanbäck, 2002; Roughgarden, 1972, 1974).

Diet indices for each site were estimated using RInSp (Zaccarelli, Bolnick, & Mancinelli, 2013) in R v 3.3.0 (R Development Core Team, 2016). Data were plotted and, if necessary, arcsine‐square root transformed to achieve normal distribution. An ANOVA with sites nested within region and habitat was run to investigate the effects of habitat and region on diet specialization. Primer v 7.0.9 (Clarke & Gorley, 2015) with the PERMANOVA add‐on package was used for all multivariate diet data analyses. Sites were nested within habitat and region, and all of these factors were treated as fixed effects. All diet data were arcsine‐square root transformed (% dry mass), and ordinations were based on Bray–Curtis similarity. Unrestricted permutations (9,999 permutations) with type III sums of squares were used to test for the significance of models.

### 1.4 Habitat coupling assessed by stable isotope analysis

Stable isotopes indicate the long‐term resource use of pelagic and littoral perch and were used to indicate habitat coupling occurring over a longer period of time (Scharnweber et al., 2016). Analyses of δ^13^C and δ^15^N on perch muscle tissue and baseline samples were done at the Stable Isotope Facility, University of California, Davis (CA), using PDZ Europa ANCA‐GSL elemental analyzer interfaced to a PDZ Europa 20–20 isotope ratio mass spectrometer (Sercon Ltd., Cheshire, UK). Samples were dried at 60°C for 48 hr, ground to a powder and weighed, and then placed into tin capsules (ca. 1 mg of tissue). Perch muscle samples were not corrected for lipid content due to their low C:N ratios (Pinnegar & Polunin, 1999; Post et al., 2007). A third of all perch were processed in duplicate, and the analytical error was 0.16‰ and 0.17‰ for δ^13^C and δ^15^N, respectively. We defined the isotopic baseline at the time of sampling for each site by isotope values of Bivalvia, Gastropoda, and zooplankton. For more details of sampling design and methods, see Supporting Information Appendix S1.

To assess the proportion of pelagic and littoral components in the diet of perch at each site, a Bayesian mixing model MixSIAR v 3.0.2 (Stock & Semmens, 2013) in R v 3.3.0 (R Development Core Team, 2016) was used, following the approach of Scharnweber et al. (2016). Fractionation factors of 0.4 ± 1.3 for δ^13^C and 3.4 ± 1.0 for δ^15^N were used to correct for trophic fractionation (Post, 2002). Prior to model runs, isotope biplots were inspected to validate the models (see Supporting Information Figure S1 for biplot of raw stable isotope values). We used Gelman–Rubin and Geweke diagnostics to check for chain convergence. Informative prior distributions were included based on a previous study of perch diet in the same study system (Bartels, Hirsch, Svanbäck, & Eklöv, 2012). We report 95% credibility intervals for all results from the Bayesian mixing model. Standard ellipse areas corrected for small sample size (SEAc) were calculated using the R package SIBER v 2.0.2 2 (Jackson, Inger, Parnell, & Bearhop, 2011). To exclude length as a factor in the stable isotope analyses, separate statistical analyses were run with the raw stable isotope values as response values and habitat and perch length as predictors. Length was not an important factor (*p* > 0.05) (see Supporting Information Appendix S1 for details and Supporting Information Figures S2 and S3 for stable isotope values vs. habitat and perch length).

### 1.5 Habitat coupling assessed by morphological and genetic analysis

Perch were photographed on their left side with a bright background and under constant light conditions. Landmark‐based thin‐plate spline analysis was used to characterize perch morphology. Eighteen landmarks were digitized from the photographs using TPS‐dig2 (Supporting Information Figure S4) (Rohlf2015a, a), and tpsRelw (Rohlf, 2015b) was used to estimate relative warp scores. These relative warp scores were used to calculate the Euclidean distance (ED) of each perch. This distance is the total vector distance between the centroid of the perch and the landmarks for the individual compared to the average perch within its site. Euclidean distances were analyzed by ANOVA in R with sites nested within region and habitat.

Body form changes were visualized using MorphoJ (Klingenberg, 2011). The digitized data from TPS‐dig2 were imported into MorphoJ and checked for outliers. The data were size corrected for body size by regressing shape scores (Procrustes coordinates) against the centroid size following the approach outlined in Klingenberg (2016). The residuals of this regression were then used for all further analysis. Canonical variate analysis (CVA) and discriminant function analysis (DFA) were used to assess the significance of shape differences (Mahalanobis distance) of perch between regions.

Fin clip DNA was extracted using a modified salting out method (Paxton, Thoren, Tengo, Estoup, & Pamilo, 1996). Nine microsatellite loci were amplified using previously developed primers: PflaL2, PflaL4, PflaL5, PflaL9, and PflaL10 (Leclerc, Wirth, & Bernatchez, 2000); SviL7 (Wirth, Saint‐Laurent, & Bernatchez, 1999); and Svi6, Svi17, and Svi18 (Borer, Miller, & Kapuscinski, 1999). See Supporting Information Appendix S1 for polymerase chain reaction (PCR) details.

PCR products were screened on an ABI3730XL and scored with GeneMarker v 2.40 (Hulce, Li, Snyder‐Leiby, & Johathan Liu, 2011). Peaks were binned automatically and manually checked. Genotyping errors due to allelic dropout and null alleles were screened using MICRO‐CHECKER v 2.2.3 (Van Oosterhout, Hutchinson, Wills, & Shipley, 2004). Linkage disequilibrium and deviations from Hardy–Weinberg's equilibrium were assessed in GENEPOP (http://genepop.curtin.edu.au) (Raymond & Rousset, 1995; Rousset, 2008), using sequential Bonferroni correction for multiple testing where appropriate. None of the nine loci showed significant allelic dropout, null alleles, linkage disequilibrium, or deviations from Hardy–Weinberg equilibrium. However, one locus (SviL7) was excluded from further analysis due to missing data (genotypes missing at 66% of perch). Of the total of 494 perch, 461 were successfully genotyped on the remaining eight loci.

To explore genetic differentiation between sites, *F*_ST_ was estimated using GenoDive v 2.0b27 (Meirmans & Van Tienderen, 2004). As long‐term isolation between sites was not expected, pairwise relatedness (r) Lynch and Ritland (1999) was also calculated using GenALEx v 6.5 (Peakall & Smouse, 2006, 2012). Mean relatedness within region was estimated in GenALEx. To further explore whether region was a barrier to gene flow, the distributions of relatedness between pairs of individuals within (e.g., pelagic east vs. pelagic east; littoral north vs. littoral north) and between (e.g., pelagic east vs. littoral north) regions were visually compared. If regions were a barrier to gene flow, a higher proportion of related individuals would be expected within rather than between regions.

## 2 RESULTS

### 2.1 Diet

**Figure 2:**
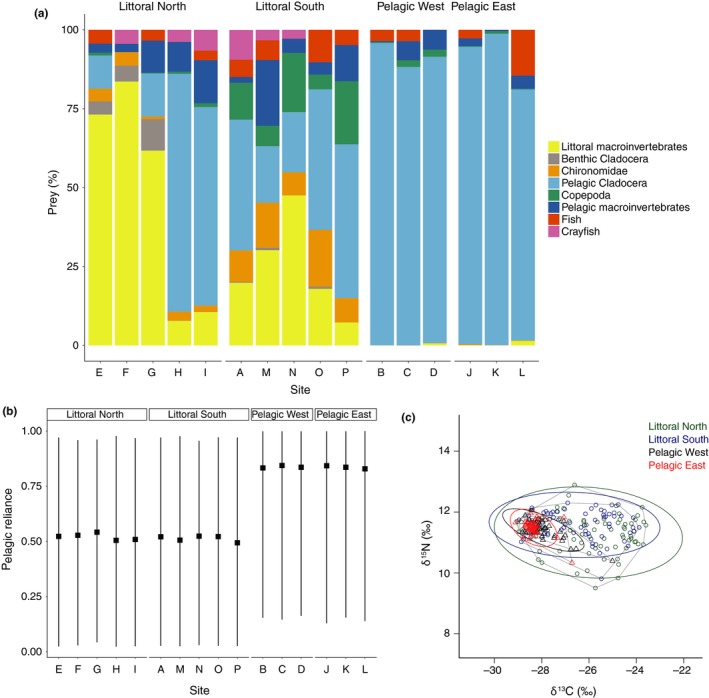
Resource usage of perch at the 16 sampling sites and four regions based on gut content and stable isotope analysis. (a) Percentage of prey types in the diet of perch at each site. Terrestrial prey was found to have been consumed by only one perch in site A (1% of diet) and was excluded. (b) 95% credibility intervals of perch for the 16 different sites, calculated with Bayesian mixing models MixSIAR v 3.0.2 (Stock & Semmens, 2013). (c) Biplot of stable carbon and nitrogen isotope ratios. Polygons illustrate the total niche width occupied by the individual perch in the four regions

Of the 494 perch stomachs examined, 80 were empty. The diet of the remaining 414 stomachs differed significantly between habitats (PERMANOVA: *Pseudo‐F* = 157.40, *p* < 0.001; Figure 2a), regions (PERMANOVA: *Pseudo‐F* = 9.77, *p* < 0.001), and sites (PERMANOVA: *Pseudo‐F* = 9.37, *p* < 0.001). There was a significant difference in diet between the littoral north and littoral south regions (post hoc pairwise comparison: PERMANOVA: *Pseudo‐F* = 3.62, *p* < 0.001), but no difference between the pelagic west and pelagic east regions (post hoc pairwise comparison: PERMANOVA: *Pseudo‐F* = 1.18, *p* = 0.24). Reliance on pelagic resources was high in perch caught in all regions, but the pelagic reliance was highest in the pelagic regions (Figure 2b).

The total niche width in isotope space was highest in the littoral north region, followed by littoral south, pelagic west, and pelagic east. Stable isotope standard ellipse areas differed among the regions, being higher in the littoral habitats indicating a wider range of resource use in these two regions (Littoral North: 3.15; Littoral South: 2.06) compared to the two pelagic regions (Pelagic West: 0.48; Pelagic East: 0.29). All ellipses showed substantial overlap, indicating common resources were used to some extent across the regions (Figure 2c).

### 2.2 Diet specialization and niche width

**Figure 3:**
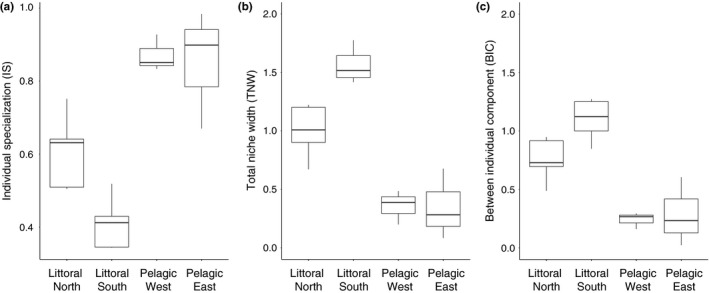
Box‐and‐whisker plots showing diet niche width metrics for each region and habitat. (a) Individual specialization (IS) ranges from 1, when all individuals are generalists and use the full range of the population's niche, to 0, when each individual uses only a single prey type and there are no overlapping diets. (b) Total niche width (TNW) quantifies the total niche width of the population. (c) Between‐individual component (BIC) is a component of TNW calculated as TNW = WIC + BIC, where WIC (within‐individual component) is the mean diet variation found within individuals, and BIC is the mean variation in resources used between individuals. Top and bottom of the boxes are first and third quartiles, the line median, with whiskers extending to ±1.5 × interquartile range

Individual specialization was significantly higher (i.e., low IS values) in the littoral sites compared to the pelagic sites (ANOVA: *F*_1,12_ = 46.07, *p* < 0.001, Figure 3a). There were also significant differences between regions (ANOVA: *F*_2,12_ = 4.87, *p *< 0.05). Post hoc Tukey's HSD tests showed that the littoral north and littoral south regions were significantly different (*p* < 0.05), with higher individual specialization (i.e., low IS values) found in littoral south than littoral north (Figure 3a), whereas the two pelagic regions did not differ (*p* = 0.99). IS did not correlate with intraspecific competition (measured as perch abundance, see Supporting Information Table S1 for data on perch abundance) within habitat (littoral: *r* = 0.56, *p* = 0.09, pelagic: *r* = −0.09, *p* = 0.92). Overall, pelagic west and pelagic east regions had low individual specialization (Figure 3a).

There were significant differences in the total niche width (TNW) between littoral and pelagic habitats (ANOVA: *F*_1,12_ = 56.46, *p* < 0.001, Figure 3b) and between regions (ANOVA: *F*_2,12_ = 4.04, *p* < 0.05). Post hoc tests showed that differences between the littoral north and south regions were marginally not significant (*p* = 0.065). The total niche width was largest in the littoral south followed by the littoral north. Total niche width was low in both pelagic regions.

There were significant differences in between‐individual component (BIC) between littoral and pelagic habitats (ANOVA: *F*_1,12_ = 44.13, *p* < 0.001, Figure 3c) and between regions (ANOVA: *F*_2,12_ = 3.99, *p* < 0.05). Post hoc tests revealed that the differences between the littoral north and south regions were marginally not significant (*p* = 0.066). The between‐individual component was highest in the littoral habitats and lowest in the pelagic habitats.

### 2.3 Morphology

**Figure 4:**
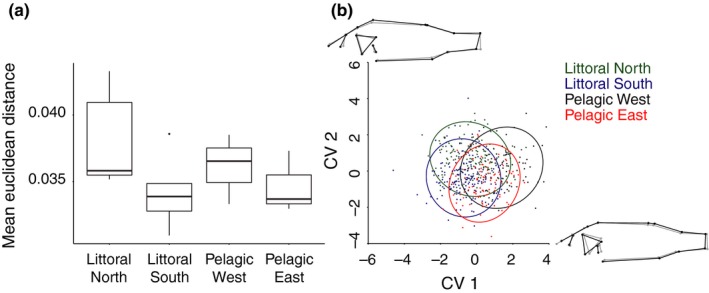
Measure of morphological variation and shape difference of perch in the four regions. (a) Box‐and‐whisker plots showing Euclidean distance (ED) for each region. Top and bottom of the boxes are first and third quartiles, the line is the median, with whiskers extending to ±1.5 × interquartile range. Dots indicate outliers, that is points >1.5 × interquartile range. (b) Canonical variate analysis (CVA) for perch morphology showing the four regions. Visualizations of perch morphology (amplified ten times) are shown on the bottom‐right and top‐left to illustrate the shape changes of the perch along CV1 and CV2 axes, respectively. The starting shape (negative values on CV axes) is shown with gray outline and the end shape (positive values on CV axes) as black outline

Group averages of morphological variation among individuals (ED) differed significantly among the four regions (ANOVA: *F*_2,476_ = 5.39, *p *< 0.001; Figure 4a). Post hoc tests showed that only the littoral north and littoral south regions were significantly different (*p* < 0.05). There was no significant difference in ED between habitats (ANOVA: *F*_1,476_ = 0.35, *p *= 0.55) or among sites (ANOVA: *F*_12,476_ = 1.53, *p *= 0.11).

Body form varied significantly among the four regions and between the habitats (permutation tests of Mahalanobis distances, Supporting Information Table S2). Compared to pelagic perch, perch from the littoral regions had a more downward positioned snout and an operculum bone that was angled in a way to indicate a downward‐pointing snout (negative CV1 values; Figure 4b). The position of the operculum bone, snout length, caudal and pectoral fin positions, and body height were the main morphological differences (Figure 4b). CV1 explained 57% of the variance in morphology, which was associated with the position of the operculum bone, snout length, pectoral fin position, and body height. CV2 explained 27% of the variance and was associated with the caudal fin shape and body height (Figure 4b).

### 2.4 Microsatellites

**Figure 5:**
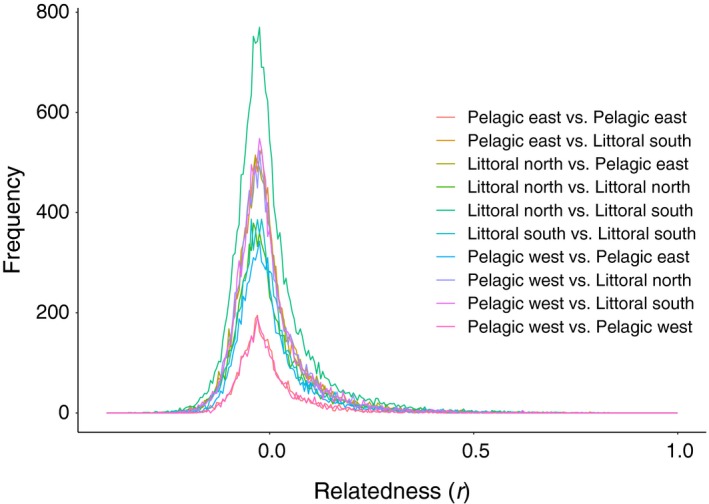
Frequency histogram of pairwise perch relatedness (r) among and within the four regions

Genetic diversity was similar in each region (Supporting Information Table S3). Within‐region relatedness ranged from −0.004 in pelagic east to 0 in littoral south (Supporting Information Figure S5). Pairwise relatedness showed no difference within or between regions (Figure 5, Supporting Information Table S3). Genetic differentiation (*F*_ST_) was very low between all pairs of regions (*F*_ST _range −0.001 to 0.003), but was significantly different between littoral south and both pelagic east (*F*_ST_ = 0.002; *p* = 0.05) and pelagic west (*F*_ST_ = 0.003; *p* = 0.024).

## 3 DISCUSSION

We show that perch display asymmetrical habitat coupling, where littoral perch clearly feed from both littoral and pelagic habitats to a higher degree than pelagic perch. Both diet and stable isotope results indicated that littoral perch include littoral and pelagic resources in their diet, while the pelagic perch were more reliant on pelagic resources only. This niche expansion of littoral predators by including pelagic prey items in their diet is consistent with general theory showing that predators normally considered as generalists, such as perch, may have a greater ability to couple habitats than more specialist predators (McCann et al., 2005; McCann & Rooney, 2009; Rooney et al., 2008). However, littoral perch also showed higher individual specialization than the pelagic perch, which is contrary to the expectation that littoral perch display higher generalist feeding behavior and stronger habitat coupling. Based on our results, we propose that habitat coupling, at least in this aquatic system, can be controlled by a balance between factors promoting (e.g., competitive release, increased foraging opportunity due to increased habitat diversity) and inhibiting habitat shifts (e.g., local adaptation leading to population differentiation). Below, we elaborate on how these factors, alone or together, can regulate habitat coupling.

Predator mobility can be driven by distinct usage of prey resources in different habitats, which in this case were the littoral and pelagic zones of a freshwater lake. Higher mobility of predators has been suggested as a mechanism behind habitat coupling (McCann et al., 2005; McCann & Rooney, 2009; Rooney et al., 2006, 2008), often not considering the potential for individual specialization within the population and the consequent implications. These previous studies have focused on whether turnover rates of different energy channels can be stabilized by mobile predators, but have not directly tested mechanisms of predator mobility in relation to resource preference and niche space (but see McMeans et al., 2016, Post, Conners, & Goldberg, 2000 for some empirical evidence as well as a discussion on resource selection and adaptive capacity in a food web). While it has been shown that the contribution of benthic resources is important for many pelagic feeding predatory fish species (Vander Zanden & Vadeboncoeur, 2002), our results emphasize that differences in the accessibility of habitat and resources can lead to differential habitat coupling. Potentially, this asymmetry in habitat coupling could be explained by the pelagic food web compartment providing essential resources for growth and reproduction that are not found in the littoral habitat (Scharnweber et al., 2016). Alternatively, population dynamics could influence the coupling of habitats. For example, high population densities can drive habitat shifts in perch where a part of the population moves from the preferred littoral habitat out to the pelagic habitat (Svanbäck & Persson, 2004). This asymmetry in migration might also skew habitat coupling since habitat and diet profitability will change as competition changes in the population (Svanbäck & Bolnick, 2005). While some studies have provided evidence for asymmetrical resource use (Bartels et al., 2012; Bartels, Hirsch, Svanbäck, & Eklöv, 2016; Knudsen et al., 2010; Scharnweber et al., 2016), these studies did not discuss the mechanistic drivers of asymmetrical habitat coupling.

We observed the highest individual specialization in littoral perch, which also had the broadest population niche. Our stable isotope findings corroborated with our diet results, indicating that littoral perch also had a much broader niche space than pelagic perch over a longer period of time. While total niche width is based on diet and only gives a snapshot estimate, SEAc is based on stable isotopes, which give longer time‐frame and integrated measurement of resource usage (Bolnick et al., 2002; Scharnweber et al., 2016). The high individual specialization among littoral individuals is contradictory to what we would expect if our observation was a result of niche expansion, where predators become more generalists by including more prey types in the diet. A previous study has shown perch to have various degrees of diet specialization as a result of different trade‐offs in foraging strategy (Svanbäck, Quevedo, Olsson, & Eklöv, 2015). Total niche width of a population can relate to niche width within individuals (within‐individual component, WIC), as well as between individuals (between‐individual component; BIC) (Bolnick et al., 2010). We show that perch in our study system had a low BIC in the pelagic habitat and a high BIC in the littoral habitat. Therefore, while littoral perch had a broader overall niche width, the littoral population was composed of relatively specialized individuals, and while we found no significant relationship between IS and intraspecific competition (perch abundance), it is possible that intraspecific competition was decreased by individuals dividing the resources among themselves within the population (Bolnick et al., 2010; Faulks, Svanbäck, Ragnarsson‐Stabo, Eklöv, & Östman, 2015b; Lister, 1976). Having more specialized individuals suggests that habitat coupling is not driven by individuals expanding their resource niche, but rather the population is expanding its niche. Niche expansion often implies that generalization occurs once individuals have shifted to the other habitat (i.e., the pelagic zone in our case). We anticipate a similar process here, but that littoral individuals switched between the pelagic and littoral habitats, which is supported by a substantial amount of pelagic resources found in the diet of littoral perch (i.e., pelagic Cladocera, Copepoda and pelagic macroinvertebrates). The conclusion that higher individual specialization leads to higher habitat coupling might seem counterintuitive if the littoral individual specialization would only consider littoral prey items. However, the specialization also includes pelagic prey, which implies that some littoral predators shifted to the pelagic zone and included pelagic prey in their diet. A similar dietary pattern among individuals in the pelagic sites was not expected, since the distance from the pelagic populations to the littoral zone was relatively large and we therefore do not expect individuals captured in the pelagic zone to easily move to the littoral zone. Conversely, at some of our littoral sites, the individuals consumed almost exclusively littoral prey items.

We observed deeper bodied fish in the littoral habitat and more fusiform fish in the pelagic habitat, supporting previous work showing habitat‐specific morphological adaptations (Day, Pritchard, & Schluter, 1994; Smith & Skúlason, 1996; Svanbäck & Eklöv, 2003). The morphological variation (ED) and SEAc results support expectations in most cases, where morphological variation was often positively correlated with diet variation (Roughgarden, 1972; Snowberg, Hendrix, & Bolnick, 2015; Van Valen, 1965) and individual specialization (Eklöv & Svanbäck, 2006). This suggests that if individuals trade‐off habitat‐specific foraging adaptations with differences in competition or predation between habitats, such adaptations might lead to constraints in habitat coupling (Eklöv & Svanbäck, 2006; Quevedo et al., 2009). However, one littoral region was seen to have a large niche space without a correspondingly high morphological variation and individual specialization (littoral south). A potential explanation for this result might be the composition and the accessibility of resources, where the range in habitat heterogeneity and presumably resource diversity in this region was lower than in the other littoral region. Lower habitat heterogeneity can also be related to higher individual specialization and lower morphological variation, as shown in an experiment that manipulated habitat accessibility (Marklund, Svanbäck, Zha, Scharnweber, & Eklöv, 2018). This raises the question of how habitat heterogeneity correlates to individual specialization and morphological variation. One possibility is that habitat heterogeneity increases resource diversity, which may counteract intraspecific competition promoting individual niche expansion. Intraspecific density did not affect individual specialization between habitats, instead it is possible that the relationship between intraspecific competition and individual specialization is modified by habitat heterogeneity in this littoral region.

Limitations in habitat coupling might also be a result of localized genetic differentiation, if local adaptation leads to constrained individual movements across habitats. While a previous study suggested that assortative mating of perch in this study lake might be taking place over small spatial scales (Bergek & Björklund, 2007), we found no evidence of assortative mating based on region, habitat, or morphology. Indeed, even though genetic differentiation has been found between perch populations from pelagic and littoral habitats, the main component of morphological adaptation is believed to be due to phenotypic plasticity (Faulks, Svanbäck, Eklöv et al., 2015a; Olsson & Eklöv, 2005; Svanbäck & Eklöv, 2006). Phenotypic plasticity might play a big role for predators to couple different habitats, especially when considering changing environmental conditions over longer timescales (Siepielski, DiBattista, & Carlson, 2009). Lower plasticity could potentially result in slower responses to changing foraging opportunities, reducing their habitat coupling ability, and also potentially affecting ecosystem stability (McCann et al., 2005; McMeans et al., 2016; Rooney et al., 2006).

The simple and straightforward hypothesis that mobile consumers have strong stabilizing effects in food webs is a very attractive one (McCann et al., 2005). However, this leans on the assumption of rapid behavioral response to fluctuating resources. Our study demonstrates that this ability may also be influenced by intraspecific niche partitioning and the degree of individual specialization of the predators (see also Knudsen et al., 2010, Quevedo et al., 2009). We show that niche expansion, where the littoral perch included more pelagic prey types, might have led to asymmetrical habitat coupling; that is, the littoral perch population coupled the littoral and pelagic habitats more than the pelagic perch population. Our study emphasizes the role of individual variability in niche and trophic traits for spatial interactions, and highlights the need for further studies that explore the relationship between evolutionary related traits and spatially related food web interactions.

## CONFLICT OF INTERESTS

We have no competing interests.

## AUTHOR CONTRIBUTION

MHKM, PE and RS designed the study. MHKM, PE, RS, KS, and YZ conducted the field work. MHKM, KS, YZ, and LF conducted the laboratory work. MHKM, LF, and MFB analyzed the data. MHKM wrote the first draft of the manuscript, all authors contributed to revisions.

## Data Availability

Data supporting this article have been deposited in Dryad with https://doi.org/10.5061/dryad.fb17m59.
