# Supplementary material for: Asymmetrical habitat coupling of an aquatic predator—The importance of individual specialization
Source: Ecol Evol. 2019 Feb 23;9(6):3405–15. doi: 10.1002/ece3.4973 (PMC6434573; doi:10.1002/ece3.4973)
Supplement: Supplementary file 1 [file ECE3-9-3405-s001.docx]

**SUPPORTING INFORMATION**

***Appendix S1***

***METHODS AND RESULTS***

*Stable isotope sampling, processing and statistical analysis*

We defined the isotopic baseline at the time of sampling for each site by isotope values of Bivalvia, Gastropoda and zooplankton. Three individuals of Gastropoda were collected from rocks from each littoral site (Site A, P, O, N, M, E, F, G, H, I) and analysed. Three replicates of zebra mussel (*Dreissena polymorpha*) were sampled from each littoral site (Site A, P, O, N, M, E, F, G, H, I) as well as samples from along the shore of a small island (ca 50 m in diameter) in the middle of the lake. This island has very little vegetation and a steep shoreline, and therefore we considered the baseline samples from this island to well represent the pelagic habitat (Sites B, C, D, J, K, L). Two replicates of zooplankton samples were collected by towing a zooplankton net (mesh size 100 μm) behind a boat in the pelagic habitat. All Bivalvia and Gastropoda were left in filtered lake water (GF/F filter) for 48 hours in the lab for gut evacuation before taking a sample of the soft tissue for drying and weighing.

To exclude the dependency of the total length of perch variable in the stable isotope analyses, separate statistical analyses were conducted with the raw stable isotope values as a response variable, habitat as a factor and perch length as a covariate. Residuals were checked for normality and an ANCOVA was run for δ^13^C. When perch length was found not to be significant (p > 0.05) the interaction was dropped and an ANOVA was run which showed a significant effect of habitat (F_1, 238_ = 109.74, p < 0.001, Appendix Figure S2a), with perch of the littoral habitats being enriched in ^13^C, but not perch length (F_1, 238_=0.53, p > 0.05, Appendix Figure S3a). δ^15^N data was not normally distributed, and therefore we ran a non-parametric Kruskal Wallis test. There was no significant difference between habitats χ^2^(1) = 1.0997, p = 0.2943 (Appendix Figure S2b) or perch length χ^2^(41) = 52.324, p = 0.1106 (Appendix Figure S3b). Therefore, length was concluded not to be an important factor in our analyses.

*Microsatellite PCR steps*

Type-it Microsatellite PCR Kits were used for PCRs (QIAGEN, California, USA). Each reaction had a total volume of 10 μL, and contained 1 μL DNA, 5 μL Type-it Multiplex PCR Master Mix (2×), 2 μL DNase/RNase-free H2O, 1 μL Q-Solution (5×) and 1 μL primer mix (0.2 μM). The thermo-cycling profile was 5 min at 95°C, 32× (30 s at 95°C, 90 s at 56°C, 30 s at 72°C), with a final elongation step of 30 min at 60°C.

**Appendix Figure S1.** Biplot for δ^13^C and δ^15^N of the raw isotope data of perch and resources used for the Bayesian mixing model. Dots represents individual perch, error bars are mean and SD for resources.****

**Appendix Figure S2.** Box-and-whisker plots showing δ^13^C (a) and δ^15^N (b) values for littoral and pelagic habitats. Top and bottom of the boxes are first and third quartiles, the line median, with whiskers extending to ±1.5 × interquartile range.

**Appendix Figure S3.** Plot showing δ^13^C (a) and δ^15^N (b) values for littoral and pelagic habitats versus perch total length (mm).

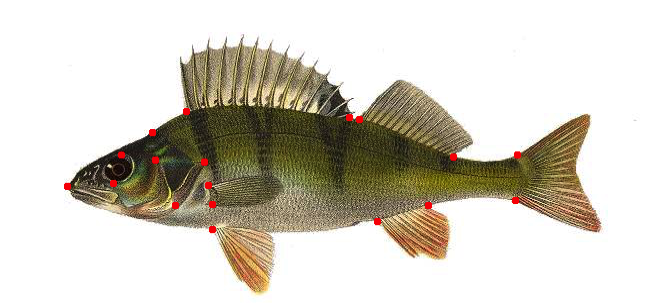


**Appendix Figure S4.** The position of the 18 digital landmarks used for perch morphology analysis.

**Appendix Figure S5**. Mean relatedness (r) within regions, based on 8 microsatellite loci, with random permutations (999) and 95% error bars determined by bootstrapping.****

**Appendix Table S1.** Abundance of perch (n), mean weight (g), sum weight (g) and mean total length (mm) for each site and region. Total abundance (n) and catch per unit effort (CPUE) for each region.

| Region | Site | n | Mean weight (g) | Sum weight (g) | Mean total length (mm) |
| --- | --- | --- | --- | --- | --- |
| Littoral North | E | 172 | 22.3 | 3831 | 99 |
|  | F | 161 | 31.2 | 5016 | 121 |
|  | G | 150 | 16.4 | 2462 | 90 |
|  | H | 418 | 16.1 | 6709 | 88 |
|  | I | 124 | 16.4 | 2032 | 104 |
|  | **Total** | **1025** | **19.6** | **20050** | **97** |
|  | CPUE | 102.5 |  |  |  |
| Littoral South | A | 71 | 50.9 | 3617 | 140 |
|  | M | 125 | 13.8 | 1731 | 88 |
|  | N | 226 | 16.9 | 3827 | 80 |
|  | O | 140 | 18.6 | 2601 | 88 |
|  | P | 312 | 12.9 | 4021 | 79 |
|  | **Total** | **874** | **18.1** | **15798** | **87** |
|  | CPUE | 87.4 |  |  |  |
| Pelagic East | J | 146 | 25.4 | 3712 | 133 |
|  | K | 69 | 28.8 | 1985 | 137 |
|  | L | 44 | 26.1 | 1147 | 133 |
|  | **Total** | **259** | **26.4** | **6844** | **134** |
|  | CPUE | 43.17 |  |  |  |
| Pelagic West | B | 72 | 26.0 | 1871 | 133 |
|  | C | 169 | 28.2 | 4766 | 137 |
|  | D | 378 | 23.6 | 8930 | 130 |
|  | **Total** | **619** | **25.1** | **15567** | **132** |
|  | CPUE | 103.17 |  |  |  |
|  |  |  |  |  |  |
| **Grand Total** |  | **2777** | **21.0** | **58258** | **105** |

**Appendix Table S2.** Results of pairwise Discriminant Function Analysis of perch morphology. Including the sample size (n), Mahalanobis Distances *D*, which describe the distance of group means in shape space, and *P* values (1000 permutation runs).

| **Comparisons** | **n** | **Mahalanobis distance *D*** | ***P*** |
| --- | --- | --- | --- |
| Pelagic East vs. Littoral North | 251 | 1.683 | < 0.0001 |
| Pelagic East vs. Littoral South | 244 | 1.624 | < 0.0001 |
| Pelagic East vs. Pelagic West | 184 | 1.679 | < 0.0001 |
| Littoral North vs. Littoral South | 307 | 1.351 | < 0.0001 |
| Littoral North vs. Pelagic West | 247 | 1.831 | < 0.0001 |
| Littoral South vs. Pelagic West | 240 | 2.274 | < 0.0001 |

**Appendix Table S3.** Genetic diversity and mean relatedness of perch in Lake Erken based on 8 microsatellite loci. Mean and (SE) over all loci for the four regions. Sample size (n), observed heterozygosity (Ho), expected heterozygosity (He), mean numbers of different alleles (Na) and mean relatedness value (r).

| **Region** | **n** | **Ho** | **He** | **Na** | **r** |
| --- | --- | --- | --- | --- | --- |
| Littoral South | 141 | 0.742 (0.052) | 0.768 (0.045) | 18.125 (3.898) | 0 |
| Littoral North | 139 | 0.760 (0.031) | 0.790 (0.036) | 17.500 (3.551) | -0.002 |
| Pelagic West | 90 | 0.782 (0.044) | 0.795 (0.037) | 16.750 (3.483) | -0.001 |
| Pelagic East | 91 | 0.809 (0.034) | 0.792 (0.035) | 16.500 (3.317) | -0.004 |

**Appendix Table S4.** F_ST_ based on 8 microsatellite loci for all pairs of sampling regions shown in the bottom matrix, and the *P* value for these comparisons in the top matrix.

| **Region** | **Littoral South** | **Pelagic West** | **Littoral North** | **Pelagic East** |
| --- | --- | --- | --- | --- |
| **Littoral South** | -- | 0.024 | 0.394 | 0.050 |
| **Pelagic West** | 0.003 | -- | 0.494 | 0.752 |
| **Littoral North** | 0 | 0 | -- | 0.878 |
| **Pelagic East** | 0.002 | -0.001 | -0.001 | -- |
